# MicroRNA-145 Regulates Chondrogenic Differentiation of Mesenchymal Stem Cells by Targeting Sox9

**DOI:** 10.1371/journal.pone.0021679

**Published:** 2011-07-20

**Authors:** Bo Yang, Hongfeng Guo, Yulan Zhang, Lei Chen, Dajun Ying, Shiwu Dong

**Affiliations:** 1 Laboratory of Biomechanics, Department of Anatomy, The Third Military Medical University, Chongqing, People's Republic of China; 2 Department of Anesthesiology, Chengdu Military General Hospital, Chengdu, People's Republic of China; 3 Department of Orthopaedics, Southwest Hospital, Chongqing, People's Republic of China; Universidade Federal do Rio de Janeiro, Brazil

## Abstract

Chondrogenic differentiation of mesenchymal stem cells (MSCs) is accurately regulated by essential transcription factors and signaling cascades. However, the precise mechanisms involved in this process still remain to be defined. MicroRNAs (miRNAs) regulate various biological processes by binding target mRNA to attenuate protein synthesis. To investigate the mechanisms for miRNAs-mediated regulation of chondrogenic differentiation, we identified that miR-145 was decreased during transforming growth factor beta 3 (TGF-β3)-induced chondrogenic differentiation of murine MSCs. Subsequently, dual-luciferase reporter gene assay data demonstrated that miR-145 targets a putative binding site in the 3′-UTR of SRY-related high mobility group-Box gene 9 (Sox9) gene, the key transcription factor for chondrogenesis. In addition, over-expression of miR-145 decreased expression of Sox9 only at protein levels and miR-145 inhibition significantly elevated Sox9 protein levels. Furthermore, over-expression of miR-145 decreased mRNA levels for three chondrogenic marker genes, type II collagen (Col2a1), aggrecan (Agc1), cartilage oligomeric matrix protein (COMP), type IX collagen (Col9a2) and type XI collagen (Col11a1) in C3H10T1/2 cells induced by TGF-β3, whereas anti-miR-145 inhibitor increased the expression of these chondrogenic marker genes. Thus, our studies demonstrated that miR-145 is a key negative regulator of chondrogenic differentiation by directly targeting Sox9 at early stage of chondrogenic differentiation.

## Introduction

Bone marrow mesenchymal stem cell (MSCs) possess the potency of self-renewal[1] and multipotential differentiation, such as chondrocytes, osteoblasts, adipocytes[2]. Additionally, MSCs present themselves as a ideal candidate for the regeneration of cartilage as they possess chondrogenic differentiation potential, are easily obtained and expanded in vitro[3], [4]. As chondrogenic differentiation rarely occurs spontaneously, the investigation of precise mechanisms for chondrogenic differentiation will ultimately contribute to a better understanding of skeletal development and diseases. Chondrogenic differentiation of MSCs is regulated by several transcription factors and growth factors, such as SRY-related high mobility group-Box (Sox) genes and the transforming growth factor (TGF)-β superfamily, respectively. In chondrogenesis, TGF-β stimulation is necessary for chondrogenesis derived from MSCs[2]. Preferentially, TGF-β1 or TGF-β3 is the most commonly used growth factor for chondrogenic differentiation. TGF-β3 enhances the early chondrogenesis of MSCs[5] and maintains a chondrogenic phenotype[6]. The cross-talk between TGF-β signal and transcription factors has an important role for the chondrogenesis of MSCs. For example, TGF-β receptor-regulated Smad3 and p300 cooperatively activate the Sox9-dependent transcription to promote the early chondrogenesis[7]. However, the precise mechanisms involved in this process still remain to be defined. Further studies are required to investigate the molecular mechanisms involved in the regulation of chondrogenesis of MSCs in response to the stimulation of TGF-β.

MicroRNAs (miRNAs) are short (21–24 nucleotides) noncoding RNAs, which are crucial regulators of protein-mediated gene expression [8]. After transcription, miRNA precursors are cleaved in the nucleus by Drosha [9], exported to cytoplasm by exportin [10], [11], cleaved by Dicer, and then inserted into an RNA-induced silencing complex (RISC) [12]. MiRNAs repress target mRNA expression by binding the miRNAs regulatory elements (MREs) located in 3′-untranslated region (3′-UTR) of mRNAs [13]. Abundant evidence suggests that miRNAs play critical roles in controlling cellular processes such as cell proliferation, apoptosis and differentiation [14], [15]. Particularly, miRNAs participate in controlling stem cells function and differentiation as evidence by the Drosha complex partners Loquacious (HIV-1 RNA binding protein 2), which is required for germ-line stem cell maintenance [16]. Additionally, Dicer-deficient mouse embryonic stem cells display severe defects in differentiation [17].

Previously, there were few studies about the mechanisms of miRNAs regulation in chondrogenic differentiation of MSCs. MiR-140 is tissue-specific for cartilage during embryonic development. It plays an important role in both cartilage development and homeostasis via regulating its downstream target genes, histone deacetylase 4 (HDAC4) and Smad3 [18], [19], [20], [21]. Lin *et al* found that miR-199* might affect its target gene Smad1 to regulate chondrogenic differentiation [22]. However, more evidences of the roles of miRNAs in regulating chondrogenic differentiation is needed.

In this study, to determine the roles of miRNAs in chondrogenic differentiation of MSCs, we focused on characterization of miR-145 whose expression level was gradually decreased during TGF-β3-induced chondrogenic differentiation of murine MSCs[23]. Other studies have identified the function of miR-145 involved in various oncogenic pathways [24], [25], [26], the differentiation of embryonic stem (ES) cells [27] and smooth muscle cells (SMCs) fate decisions [28], [29], [30]. Here, we show that miR-145 has a complementary role in suppressing chondrogenic differentiation of the murine embryonic mesenchymal cell line C3H10T1/2 cells. Through dual-luciferase reporter gene assay and gain- or loss-of-function experiments, we demonstrated that miR-145 can target and suppress the expression of SRY-related high mobility group-Box gene 9 (Sox9). Sox9 is a master positive regulator of chondrogenesis and regulation of Sox9 may affect chondrogenic differentiation of MSCs [31], [32], [33], [34]. Importantly, over-expression or suppression of miR-145 resulted in inhibiting or promoting chondrogenic differentiation, respectively. Our results suggest that miR-145 acts as a key mediator to antagonize early chondrogenic differentiation via attenuating the effect of transcription factor Sox9.

## Results

### MiR-145 is down-regulated during TGF-β3-induced murine MSCs chondrogenic differentiation

**Figure 1:**
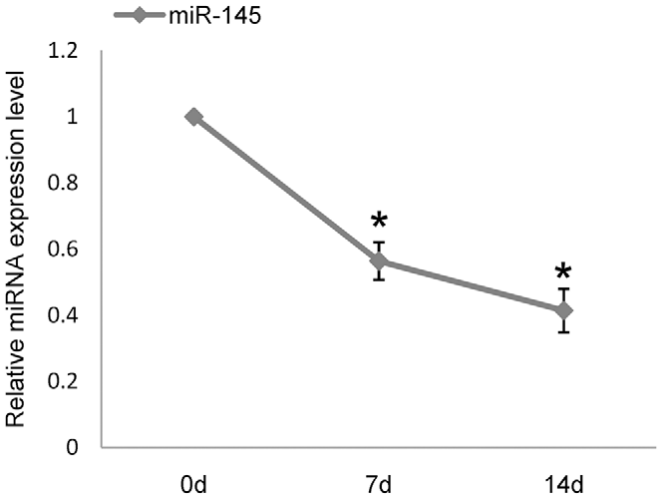
Differential expression of miR-145 during chondrogenic differentiation of murine MSCs. Down-regulated expression of miR-145 in murine MSCs induced by TGF-β3 was identified by qRT-PCR at different stage of chondrogenic differentiation (0 d, 7 d, 14 d). The relative expression level of miR-145 in untreated MSCs (0 d) was set to one, as control. Three independent cell culture experiments were done and data was represented as mean±sd. *, *p*<0.05, when compared with control.

In our previous study, miRNA microarray technology was applied to detect miRNAs expression profiles of three different stages during chondrogenic differentiation, including murine MSCs, chondrogenic induction at 7 d, and 14 d after TGF-β3 treatment[23]. The expression of miR-145 significantly decreased during chondrogenic differentiation. Subsequently, we performed bioinformatic analyses to predict the target genes of miR-145 using Pictar [35] and Targetscan [36]. Noticeably, we found Sox9 was the potential target gene regulated by miR-145. According to the primary role of Sox9 in the process of MSCs differentiation into chondrocytes, we hypothesized that Sox9 may be inhibited by miR-145, which prevents MSCs from differentiating into chondrocytes. Furthermore, down-regulation of miR-145 may act as positive effect on chondrogenic differentiation. Thus qRT-PCR assay was performed to validate the expression pattern of miR-145 in this study. The results confirmed that miR-145 gradually decreased in MSCs, which were induced by TGF-β3 (Figure 1).

### MiR-145 targets Sox9 by binding 3′-UTR of Sox9 mRNA

**Figure 2:**
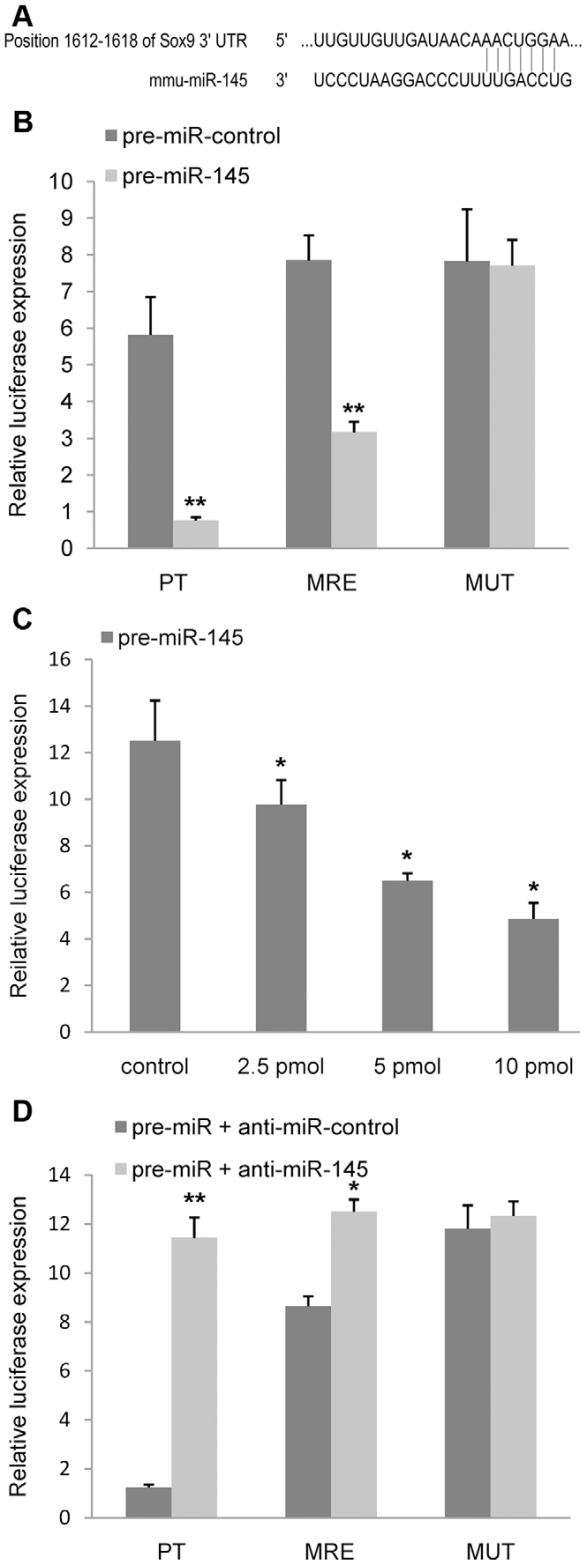
Mir-145 directly targets Sox9. (A) A schematic shows that the 5′ end of miR-145 contains a sequence complementary to the specific miRNA binding site within the 3′-UTR of Sox9 mRNA. (B) Pre-miR-145 or its negative control was co-transfected with the specific pMIR-REPORT construct containing a consensus miR-145-binding site (pMIR-PT) into HEK293 cells. Pre-miR-145 or its negative control was co-transfected with pMIR-MRE into HEK293 cells. pMIR-PT acts as a positive control. (C) HEK293 cells were co-transfected with pMIR-MRE together with varying amounts of pre-miR-145, as indicated. Statistical comparisons were made between multiple groups by ANOVA. (D) Anti-miR-145 or its negative control was co-transfected with either pre-miR-145 together with pMIR-MRE or pre-miR-145 together with pMIR-MUT into HEK293 cells. pMIR-PT acts as a positive control. All cells (B, C, D) were harvested at 48 h after transfection, and then luciferase activities were measured and normalized to the phRL-TK activities. Three independent transfection experiments were done and data was represented as mean±sd. *, *p*<0.05, **, *p*<0.01, when compared with control.

MiRNAs inhibit mRNA expression by binding the MREs located in 3′-UTR of target mRNA. The Sox9 3′-UTR contains one putative miR-145 seed site which is bound with imperfect complementation (Figure 2A). To determine if miR-145 targets Sox9, we applied the luciferase report gene assay using the pMIR-REPORT Luciferase reporter. Firstly, we constructed a reporter vector containing a consensus miR-145-binding site within the 3′-UTR (pMIR-PT, Table 1) as a positive control. After we co-transfected this reporter plasmid into HEK293 cells with pre-miR-145 or its control pre-miR, we found that luciferase expression significantly decreased in the HEK293 cells transfected with pre-miR-145 (Figure 2B). These data indicate miR-145 can suppress expression of transcripts containing an exact miR-145-binding site by our luciferase reporter assay. Next, we created specific reporter vectors, which contained either two copies of the endogenous MREs found in the Sox9 mRNA 3′-UTR (pMIR-MRE, Table 1) or corresponding two copies of the MREs with a scrambled seed sequence (pMIR-MUT, Table 1). Previous studies have proven that such reporter constructed with multiple MREs is an available method for the identification of miRNAs target genes [37], [38], [39], [40]. Co-transfection of pre-miR-145 with wild-type pMIR-MRE resulted in a suppression of luciferase gene expression, but co-transfection of pre-miR-145 and mutant pMIR-MUT did not (Figure 2B). Furthermore, the suppression effect occurred in a dose dependent manner (Figure 2C). In addition we found that anti-miR-145 could overcome the suppression effect when it was co-transfected with pMIR-MRE and pre-miR-145. However, the effect of anti-miR-145 was abolished when pMIR-MUT was used instead of pMIR-MRE (Figure 2D). Taken together, our data suggest that miR-145 could target Sox9 by binding the MREs within the Sox9 mRNA 3′-UTR.

**Table 1:** Synthesized oligonucleotides sequences for generation of luciferase reporter constructs.

Plasmid	Oligonucleotides sequences
pMIR-PT F	5′-CTAGTAAGGGATTCCTGGGAAAACTGGACA-3′
pMIR-PT R	3′-ATTCCCTAAGGACCCTTTTGACCTGTTCGA-5′
pMIR-MRE F	5′-CTAGTTTGTTGTTGTTGATAACAAACTGGAAAtagctaaTTGTTGTTGTTGATAACAAACTGGAAAA-3′
pMIR-MRE R	3′-AAACAACAACAACTATTGTTTGACCTTTatcgattAACAACAACAACTATTGTTTGACCTTTTTCGA-5′
pMIR-MUT F	5′-CTAGTTTGTTGTTGTTGATAACAACAGAATGAtagctaaTTGTTGTTGTTGATAACAACAGAATGAA-3′
pMIR-MUT R	3′-AAACAACAACAACTATTGTTGTCTTACTatcgattAACAACAACAACTATTGTTGTCTTACTTTCGA-5′

The sequences of linker are in lowercase.

### MiR-145 inhibits Sox9 expression at early stage of chondrogenic differentiation

**Figure 3:**
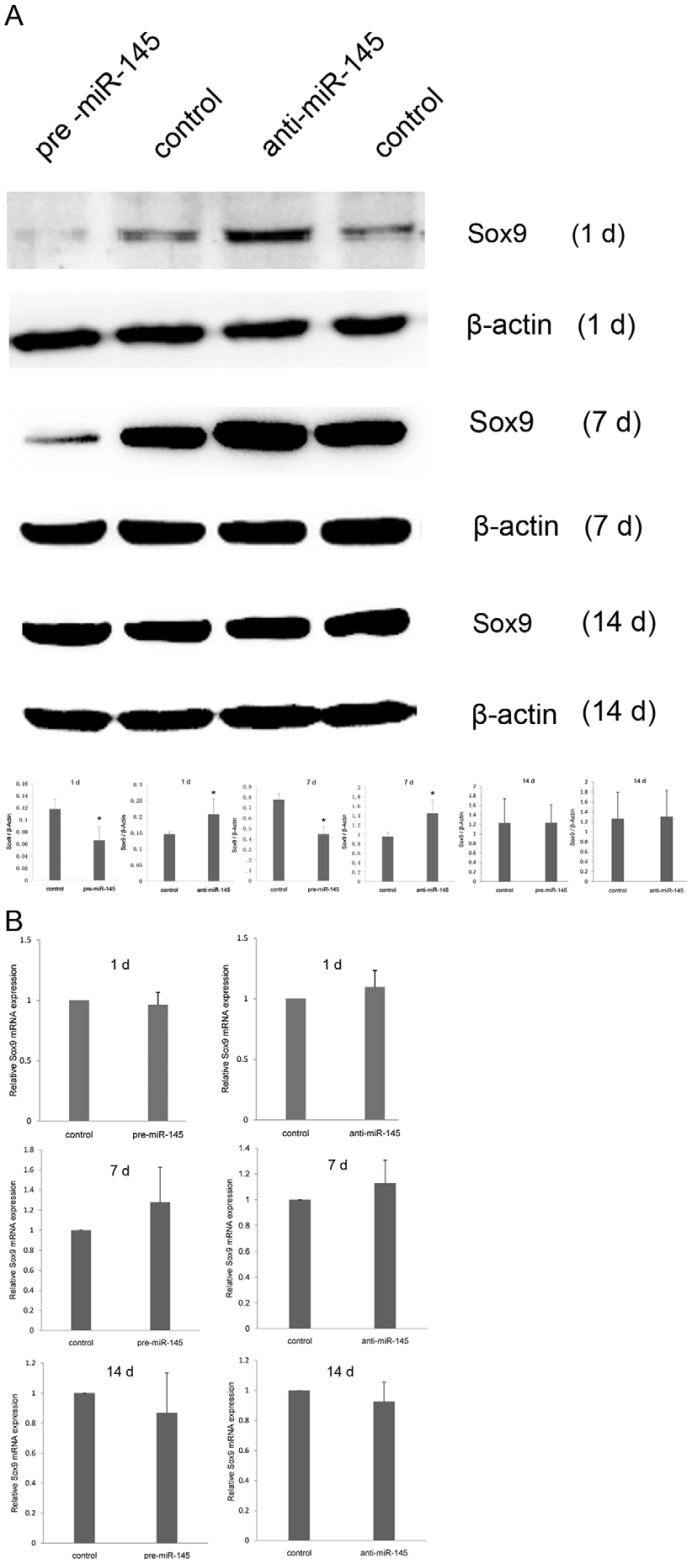
Mir-145 represses the expression of Sox9 at protein level in early stage of chondrogenic differentiation. (A) C3H10T1/2 cells were transfected with pre-miR-145, anti-miR-145 or their control individually. After induced by TGF-β3 for 24 h, 7 d and 14 d, the cells were harvested for measurement of Sox9 protein expression using Western blot. β-actin acts as an internal control. Quantitation of the Sox9 protein level was performed using Quantity One software. The result is shown in the below panels. (B) C3H10T1/2 cells were transfected with pre-miR-145, anti-miR-145 or their control, and then mRNA level of Sox9 were measured using qRT-PCR at 24h. β-actin acts as an internal control in qRT-PCR analysis. The relative expression level of Sox9 mRNA in cells transfected with control oligonucleotide was set to one, as control. Three independent experiments were done and data was represented as mean±sd.

The different degree of complementary between miRNA and its target mRNA probably determines that miRNA repress target mRNA through two distinct pathways. MiRNA suppresses mRNA translation bearing imperfect complementary target sequences and degrades mRNA bearing perfect complementary target sequences[41], [42], [43]. Computational algorithms predicted that miR-145 would bind to the Sox9 3′-UTR with imperfect complementation, suggesting that it may not result in Sox9 mRNA cleavage. To demonstrate whether miR-145 acts as attenuator of Sox9 protein expression, we transfected C3H10T1/2 mesenchymal stem cells with either pre-miR-145 or anti-miR-145 for 24 h, and then exposed the transfected cells to the chondrogenic differentiation medium primarily consisting of TGF-β3. We firstly performed the non-transfected control in preliminary experiment for optimizing the condition of transfection with miRNAs. At the optimal condition of transfection, there is no significant difference of Sox9 protein expression between tansfected group and non-transfected control (Figure S1). As expected for the mechanisms of miRNAs regulation, measured by western blot assay, Sox9 protein level was notably decreased in the cells of miR-145 over-expression and increased in the cells of miR-145 suppression at 1 d and 7 d but not at 14 d by TGF-β3 treatment (Figure 3A). However, qRT-PCR analysis showed no significant change in Sox9 mRNA level in both transfected cells (Figure 3B-). Thus, these data demonstrate miR-145 inhibits Sox9 protein expression but not mRNA levels in mesenchymal stem cell line at early stage of chondrogenic differentiation.

### MiR-145 inhibits early chondrogenic differentiation

**Figure 4:**
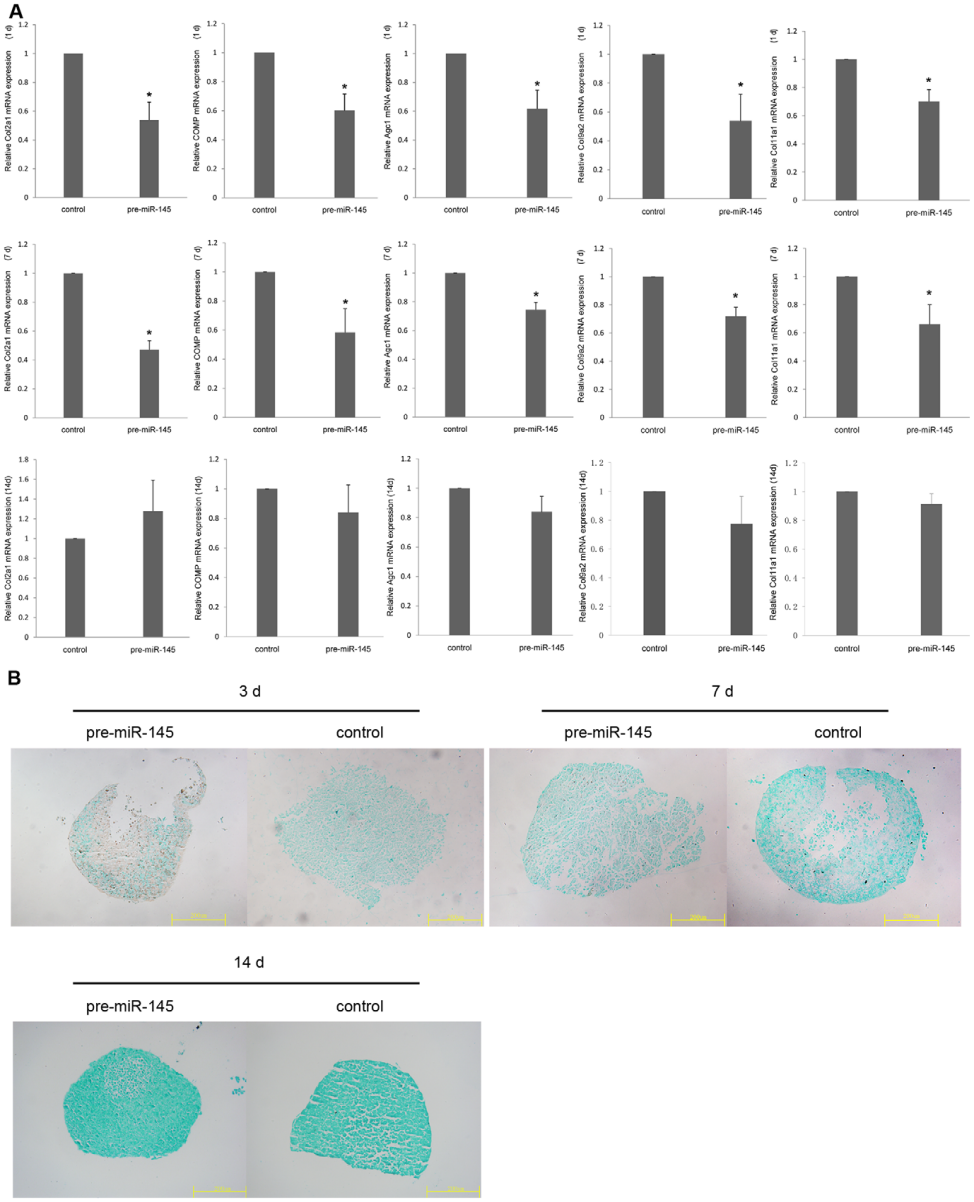
Over-expression of mir-145 inhibits early chondrogenic differentiation of C3H10T1/2 cells. C3H10T1/2 cells were transfected with pre-miR-145 or its control respectively. (A) After 24 h, 7 d and 14 d of treatment with TGF-β3, all of cells were lysed and then the expression of chondrogenic differentiation markers, such as Col2a1, COMP, Agc1, Col9a2 and Col11a1, were measured via qRT-PCR. The relative expression level of mRNA in cells transfected with control oligonucleotide was set to one, as control. (B) After 3 d, 7 d and 14 d of treatment with TGF-β3, all of pellets were measured by alcian blue staining. Three independent experiments were done and data was represented as mean±sd. *, *p*<0.05, when compared with control.

**Figure 5:**
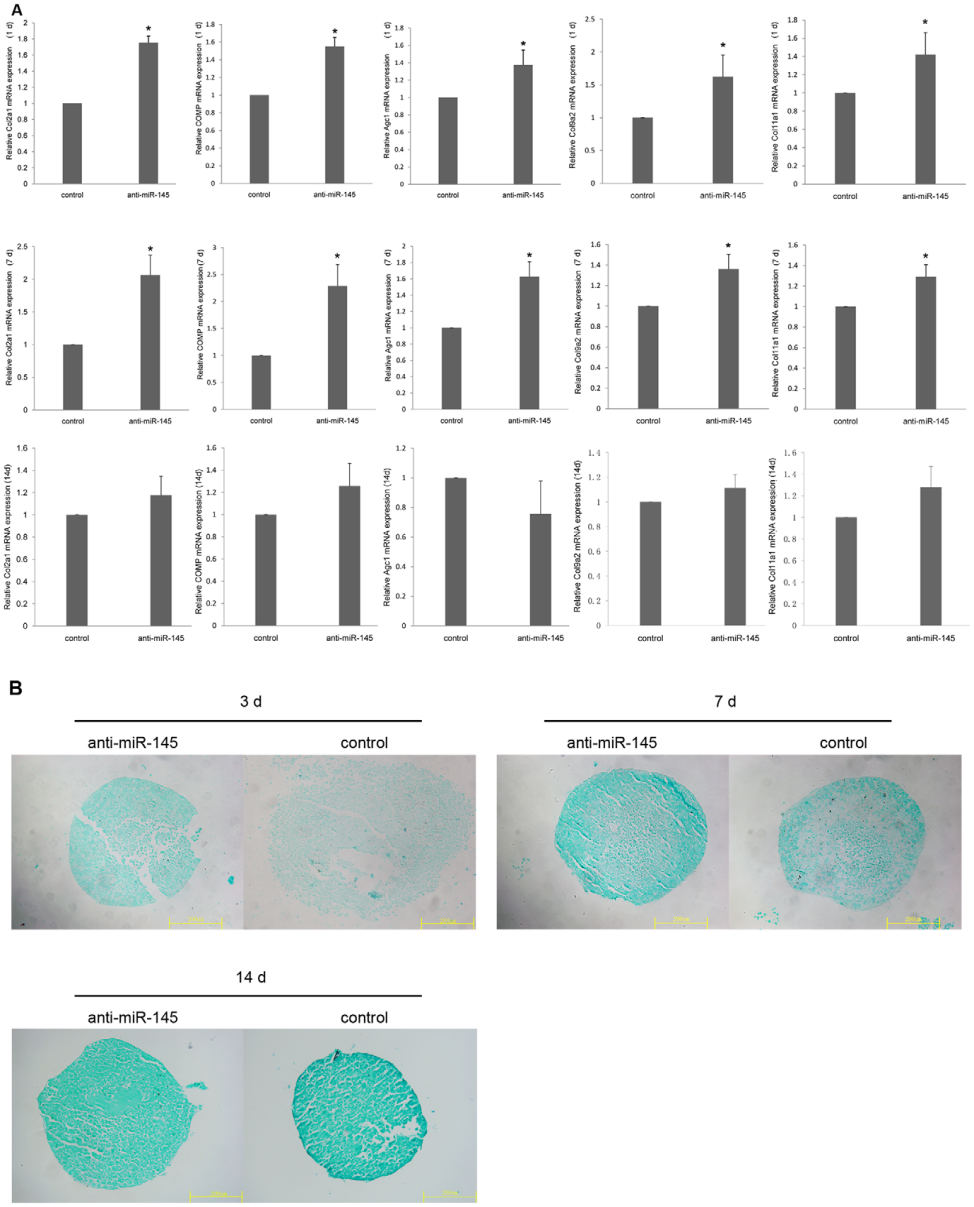
Suppression of mir-145 enhances early chondrogenic differentiation of C3H10T1/2 cells. C3H10T1/2 cells were transfected with anti-miR-145 or its control respectively. (A) After 24 h, 7 d and 14 d of treatment with TGF-β3, all of cells were lysed and then the expression of chondrogenic differentiation markers, such as Col2a1, COMP, Agc1, Col9a2 and Col11a1, were measured via qRT-PCR. The relative expression level of mRNA in cells transfected with control oligonucleotide was set to one, as control. (B) After 3 d, 7 d and 14d of treatment with TGF-β3, all of pellets were measured by alcian blue staining. Three independent cell culture experiments were done and data was represented as mean±sd. *, *p*<0.05, when compared with control.

To explore whether miR-145 has an effect on chondrogenic differentiation, we transfected either pre-miR-145 or anti-miR-145 into C3H10T1/2 cells. After induction of chondrogenic differentiation by medium containing TGF-β3 for 1 d and 7 d, qRT-PCR analysis of C3H10T1/2 cells transfected with pre-miR-145 showed a significant decrease in the mRNA expression levels of chondrogenesis markers including Col2a1, Agc1, COMP , Col9a2 and Col11a1 (Figure 4A). Moreover, alcian blue staining intensity were decreased following pre-miR-145 treatment for 3 d and 7 d (Figure 4B). These results reveal that miR-145 over-expression inhibits early chondrogenic differentiation. Inhibition of endogenous miR-145 expression in C3H10T1/2 cells by transfection of anti-miR-145, under the same induction conditions as above, resulted in enhancing chondrogenic differentiation as shown by a significant increase in chondrogenesis markers at mRNA level (Figure 5A) and alcian blue staining intensity (Figure 5B). The results of qRT-PCR and alcian blue staining have shown that modulation of miR-145 effected the expression of genes and GAGs related to chondrocyte after induced for 7 d. However, the same effect did not last for 14 d (Figure 4, 5). Collectively, our data demonstrate that miR-145 act as a key negative regulator of early chondrogenic differentiation.

### Mir-145 has no influence on mRNA expression of C/EBPβ and C/EBPδ

**Figure 6:**
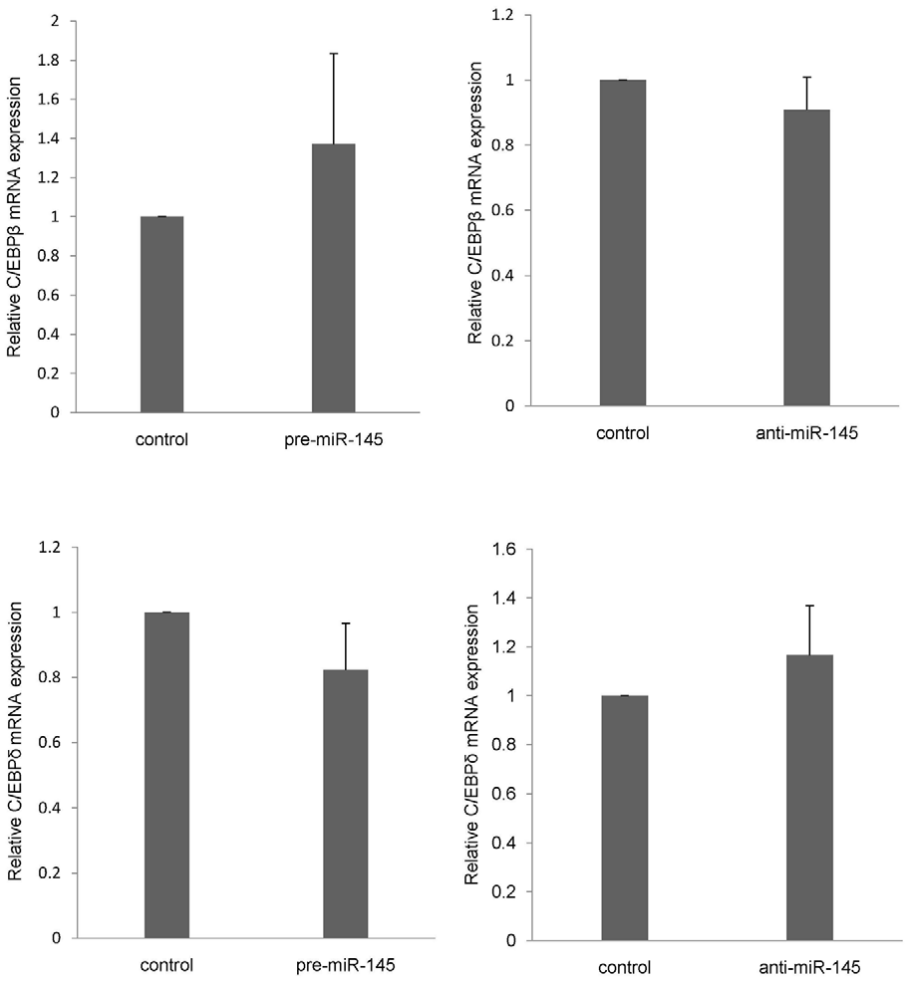
Mir-145 has no influence on mRNA expression of C/EBPβ and C/EBPδ. C3H10T1/2 cells were transfected with pre-miR145, anti-miR-145 or its control, respectively. After 24 h of treatment with TGF-β3, all of cells were lysed. The expression of C/EBPβ and C/EBPδ were measured via qRT-PCR. The relative expression level of mRNA in cells transfected with control oligonucleotide was set to one, as control. Three independent cell culture experiments were done and data was represented as mean±sd. *, *p*<0.05, when compared with control.

To investigate whether the regulation effect of miR-145 mediated by Sox9 is specific to chondrogenesis, we measured the mRNA expression level of other Sox9 non cartilage target genes. Sox9 directly binds to the promoter regions of C/EBPβ and C/EBPδ to suppress their promoter activity, preventing adipocyte differentiation[44]. Our results showed that miR-145 has not effected the mRNA expression of C/EBPβ and C/EBPδ after induction of chondrogenic differentiation (Figure 6). It suggests that the effect which miR-145 regulate chondrogenic differentiation of MSCs mediated by Sox9 in response to TGF-β3 is a specific influence on genes associated with chondrogenesis.

### Mir-145 has no influence on proliferation of C3H10T1/2 cells

**Figure 7:**
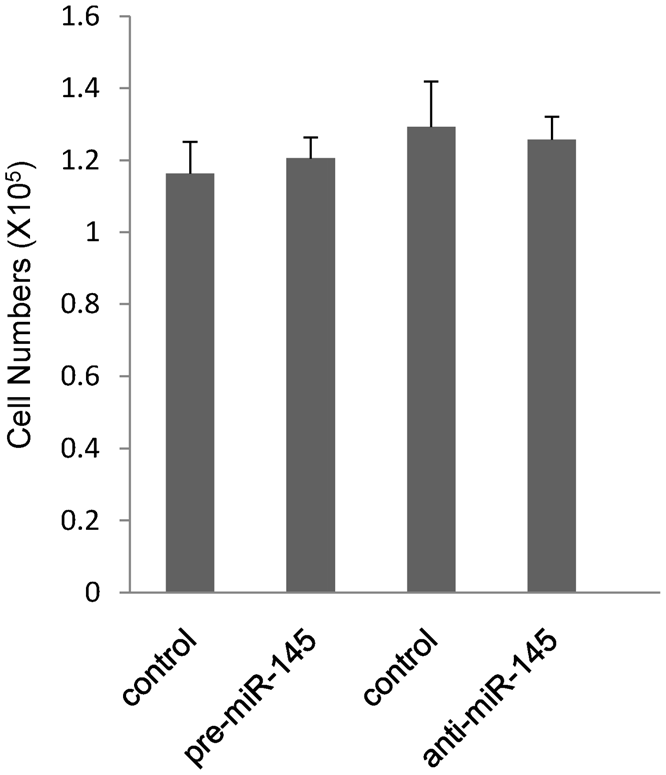
Mir-145 has no influence on proliferation of C3H10T1/2 cells. C3H10T1/2 cells were transfected with pre-miR145, anti-miR-145 or its control, respectively. After 24 h of treatment with TGF-β3, all of pellets were trypsinized and directly counted in triplicate using a hemacytometer. Data was represented as mean±sd.

Skelotogenesis is dependent upon proliferation of progenitor cells that is followed by differentiation. To investigate whether miR-145 inhibition that leads to an increase in Sox-9 protein expression affects cell proliferation, we assessed the proliferation of C3H10T1/2 cells by direct cell counting. However, treatment of C3H10T1/2 cells with either pre-miR-145 or anti-miR-145 inhibitor did not significantly effect the cell proliferation (Figure 7).

## Discussion

MiRNAs, as endogenous small molecular regulators of gene expression, play critical roles in stem cell function [45], [46] and provide new insight into precisely controlling cell fate decisions. In this study we identified that miR-145 can suppress TGF-β3-induced chondrogenic differentiation of MSCs by directly targeting Sox9, the key transcription factor for chondrogenesis, at the post-transcriptional level. After TGF-β3 treatment, the decrease in expression of miR-145 allows for the positive effect on chondrogenesis of Sox9, therefore facilitating chondrogenic differentiation.

Our microarray and qRT-PCR data demonstrate the gradually decreased expression of mature miR-145 during TGF-β3-induced chondrogenic differentiation of murine MSCs. The similar differential expression patterns of miR-145 had been described in previous studies. MiR-145 is decreased significantly in BMP-2-induced C2C12 cells detected by microarray analysis [22]. Additionally, compared with the expression level in human cartilage chondrocytes, miR-145 is also decreased in dedifferentiation of chondrocytes as validated by microarray and qRT-PCR analysis [47]. Therefore, with the combination of our microarray and qRT-PCR data, miR-145 emerged as a candidate with significant potential to participate in the regulation of chondrogenic differentiation. Although miR-145 has been known to be involved in vascular pathogenesis via maintaining the differentiation status of smooth muscle cells (SMCs) and regulation of embryonic stem (ES) cells self-renewal program [27], [48], we have identified another complementary function to inhibit Sox9-mediated chondrogenesis.

Using Pictar and Targetscan online software for prediction, we found several potential target genes of miR-145 closely related with chondrogenesis, specifically transcription factor Sox9 which is the primary determinant during the early stages of chondrogenesis. Pre-chondrogenic mesenchymal cells with a homozygous deletion of Sox9 are excluded from aggregated wild-type cells in mesenchymal condensation and cannot express chondrocyte-specific matrix genes such as Col2a1, Col9a2, Col11a2, and Agc1 [49]. Our luciferase reporter analysis showed that both exogenous miR-145 and anti-miR145 inhibitor regulated expression of luciferase when miR-145 MREs from Sox9 3′-UTR was fused to the luciferase and the effect lost when the scrambled MREs sequence was used. Furthermore, the magnitude of suppression was exhibited in a dose-dependent manner. These results suggest that miR-145 may regulate Sox9 expression by binding the MREs within the Sox9 3′-UTR and prompt us to investigate whether miR-145 effects chondrogenesis via targeting Sox9.

Consistent with the important role of Sox9 in regulating chondrogenesis, our results indicate that over-expression of miR-145 via pre-mR-145 precursor resulted in inhibition of C3H10T1/2 mesenchymal stem cells chondrogenic differentiation, as reflected by a decrease in the expression of Sox9 at the post-transcriptional level and a decrease in the mRNA expression of early chondrogenic markers including Col2a1, Col9a2, Col11a1 and Agc1. Accompanied with the decrease of Sox9, COMP that it expresses predominantly in cartilage and is activated by Sox9 binding its promoter [50], was also reduced significantly. In contrast, inhibition of miR-145 enhanced chondrogenic differentiation as evidenced by the remarkably increased expression of Col2a1, Col9a2, Col11a1, Agc1 and COMP mRNAs. Both in over-expression and suppression of miR-145 experiments, we found Sox9 differential expressed at the post-transcriptional level but not at the mRNA level. It reveals that miR-145 repressed Sox9 expression via its binding with imperfect complementation to the Sox9 mRNA 3′-UTR through translational inhibition. Furthermore, mir-145 has no effect on expression of C/EBPβ and C/EBPδ at mRNA levels in the chondrogenic differentiation. The results suggest that miR-145 has a specific influence on genes associated with chondrogenesis in response to TGF-β3. Taken together, miR-145 represents a key regulator for suppressing the chondrogenic phenotype in C3H10T1/2 cells by its ability to directly target Sox9 and probably be responsive to TGF-β3. However, we can not find the certain evidence that miR-145 would effect on the terminal differentiation of chondrogenesis by a long-time effect (for 14 d). It is maybe due to the transient transfection of miRNAs. On basis of current results, we only demonstrate miR-145 can regulate chondrogenesis at early stage.

Because the condensation and proliferation of mesenchymal cells is the initial step of chondrogenesis and skelotogenesis, we used directly counting cells numbers from micromass pellets to detect the proliferation of C3H10T1/2 cells which were transfected with pre-miR-145 or anti-miR-145, respectively. Unexpectedly, there is no significant difference between pre-miR-145 or anti-miR-145 treatment pellets and their control treatment pellets. It suggest that the effect of miR-145 regulating chondrogenic differentiation depends on Sox9-mediated the promotion of its target genes associated with cartilage, such as Col2a1, Agc1 and COMP, *etc.*.

An important feature of miRNAs is that one miRNA can regulate many target genes owing to the short seed match and imperfect base-pairing between miRNAs and their targets. Accordingly, miR-145 may regulate the process of chondrogenic differentiation by suppressing other target genes besides Sox9. There are a few target genes correlated with chondrogenesis predicted by combination of Pictar and Targetscan, including Smad3, type-I activin receptor (ACVR1B also known as ALK4) and type-II activin receptor (ACVR2A). Smad3 has been confirmed as a positive mediator of TGF-β-induced chondrogenesis [51]. ACVR2A can be bound by activins, which belongs to the TGF-β superfamily of structurally related signaling proteins, leading to recruitment and phosphorylation of the ACVR1B. This complex, containing activins, goes on to recruit the R-Smads, Smad2 or Smad3 [52] thereby involving the regulation of chondrogenesis. Thus, miR-145 may regulate chondrogenesis by repressing not only Sox9 but also other genes.

In conclusion, our studies demonstrate that miR-145 is decreased during TGF-β3-induced chondrogenic differentiation of murine MSCs. The attenuation of miR-145 expression positively regulates its direct target gene Sox9, and results in promoting chondrogenic differentiation of mesenchymal stem cell line. Our findings indicate that miR-145 plays a key role in chondrogenesis and may provide a novel mechanism in miRNA-mediated regulation of chondrogenic differentiation of MSCs.

## Materials and Methods

### Cell Culture

Isolation of murine MSCs from bone marrow is previously described [23], [53]. Briefly, mouse tibia and femur marrow cavity was rinsed by the syringes containing the medium under sterile conditions, which composed of low glucose DMEM (L-DMEM), 10% fetal bovine serum (FBS, Hyclone), 100 units/ml penicillin and 100 µg/ml streptomycin (Sigma). The rinsed solution was filtered through a 70 mm filter mesh and incubated at 37°C with 5% CO_2_ in a humidified incubator. Culture medium was replaced after 3 h, and every 8 h repeated this step in the following 72 h. Thereafter, fresh medium was replaced every 3 d until the cells reached 90% confluence. Cells were passaged by 0.25% trypsin (Hyclone) for 2 min at room temperature. The fourth generation cells were used for the following experiments. The Balb/c mice (six to eight weeks of age) were obtained from Institute of Animal, the Third Military Medical University (Chongqing, China). The Southwest institutional Animal Care and Use Committee at the Third Military Medical University approved all animal protocols. C3H10T1/2 mesenchymal stem cells and HEK293 cells (ATCC) were cultured in DMEM-F12 (Hyclone) containing 10% FBS. The culture medium was replaced every 2–3 d.

### Transfection assay

To demonstrate the functional relevance of miR-145, pre-miR-145 (a final concentration of 50 nM), anti-miR-145 (a final concentration of 150 nM) or their negative controls were transfected, respectively, into C3H10T1/2 cells in 6-well pellets (10^5^ cell per well) with 5 µl siPORT NeoFX transfection agent (Ambion) following the manufacturer's instructions. After incubation for 24 h, the transfected cells were trypsinized and subjected to the chondrogenic differentiation assay. After indicated time points, the cells were harvested for mRNA and protein analysis.

### Chondrogenic Differentiation Assay

Before chondrogenic differentiation, C3H10T1/2 cells ready for gain- or loss-of-function analysis, were transfected with pre-miR-145, anti-miR-145 or their negative controls. MSCs ready for miR-145 expression analysis were directly induced to chondrogenic differentiation. After transfection and incubation for 24 h, high density micromass cultures were treated as previously described [22]. The cells were trypsinized by 0.25% trypsin and modulated at a density of 10^7^ cells/mL. 10 µl of the suspension was placed into the center of each well on a 12-well plate (Corning). After incubation for 2 h at 37°C and 5% CO2, wells were flooded with 1 mL chondrogenic differentiation medium (Cyagen).The chondrogenic differentiation medium composed of dexamethasone, ascorbate, ITS+ Supplement, sodium pyruvate, proline and TGF-β3 was replaced every 2 d.

### Quantitative RT-PCR Analysis

To validate the expression pattern of miR-145, which emerged in the microarray results, qRT-PCR was performed. U6 acted as an internal control. Total RNA was used to generate cDNA by MasterAmp RT-PCR kit (Epicentre) according to manufacturer's instructions. The RT-PCR primers are as follows- U6: 5′-CGCTTCACGAATTTGCGTGTCAT-3′; miR-145: 5′-GTCGTATCCAGTGCGTGTCGTGGAGTCGGCAATTGCACTGGATACGACAGGGATT-3′. The cycle parameters for the RT reaction were 16°C for 30 min, 42°C for 40 min, and 85°C for 5 min, in a final volume of 20 µl. Subsequently, the synthesized cDNA was used for real-time quantitative PCR with SYBR Green (Invitrogen) with the amplification parameters: 95°C for 3 min, followed by 40 cycles of 95°C for 15 s, 60°C for 20 s, 72°C for 20 s, 78°C for 20 s. The primers of real-time PCR are as follows- U6 forward: 5′-GCTTCGGCAGCACATATACTAAAAT-3′, U6 reverse: 5′-CGCTTCACGAATTTGCGTGTCAT-3′; miR-145 forward: 5′-GGTCCAGTTTTCCCAGG-3′, miR-145 reverse: 5′-CAGTGCGTGTCGTGGAGT-3′.

To determine the expression levels of Sox9, Col2a1, Agc1 and COMP, total RNA was performed RT-PCR using the Rever Tra Ace-a -First Strand cDNA Synthesis Kit (Toyobo) followed by real-time quantitative PCR with SYBR Green. β-actin acted as an internal control. The cycle parameters for the RT reaction were 42°C for 10 min, 30°C for 20 min, and 99°C for 5 min. Next, a reaction mixture (Promega) containing the SYBR Green and the appropriate primers was added to a 0.2 ml MicroAmp (ABI), together with 2 µl of cDNA template, for a final reaction volume of 20 µl. The amplification parameters were 94°C for 5 min, followed by 30 cycles of 94°C for 30 s, 57°C for 30 s, 72°C for 30 s. The primers of real-time PCR are as follows-β-actin forward: 5′-GATCTTGATCTTCATGGTGCTAG-3′, Col2a1 reverse: 5′-TTGTAACCACCTGGGACGATATGG-3′; Col2a1 forward: 5′-CCCGCCTTCCCATTATTGAC-3′, Col2a1 reverse: 5′-GGGAGGACGGTTGGGTATCA-3′; Agc1 forward: 5′-CGCCACTTTCATGACCGAGAGAC-3′, Agc1 reverse: 5′-CCCCTTCGATAGTCCTGTCATTC-3′; COMP forward: 5′-GCCGCCTGGGTGTCTTCTGCTTC-3′, COMP reverse: 5′-CCCCCACACACACACCCGTAGC-3′; Sox9 forward: 5′-GGGGGTGAGCTTTGATTAATTC-3′, Sox9 reverse: 5′-GGGATTTAAGGCTCAAGGTGTTT-3′; Col9a2 forward: 5′- AGGGGTTGGGGGAGGGAGAG-3′, Col9a2 reverse: 5′- GGCATGGGTGAGGGGTAGTTGGT-3′; Col11a1 forward: 5′- GGCCAAAGGAGAAACCAGGAAG-3′, Col11a1 reverse: 5′- GGGCAGAGGCAGTCAGGAGCT-3′; C/EBPβ forward: 5′- AGGGGGTGGAAGAGAGCTGG-3′, C/EBPβ reverse: 5′- GGCCGTGCAGATCAGGGAAG-3′; C/EBPδ forward: 5′- TGGACAAGCTGAGCGACGAGTAC-3′, C/EBPδ reverse: 5′- CGCGACAGCTGCTCCACCTTC-3′. All real-time quantitative PCR reactions were performed in the PCR System 7500 (ABI). Data were analyzed using the 2-δδCt method.

### Western Blot Analysis

The cell lysates from micromass cultures of C3H10T1/2 cells transfected with pre-miR-145 or anti-miR-145 were extracted with lysis buffer containing 50 mM Tris (pH 7.6), 150 mM NaCl, 1% TritonX-100, 1% deoxycholate, 0.1% SDS, 1 mM PMSF and 0.2% Aprotinin (Sigma). After we measured the protein concentration, the equal protein samples were mixed with 5× sample buffer (Beyotime) and boiled. The samples were resolved by 10% SDS-PAGE gel and transferred on PVDF membrane (Millipore) by using the semi-dry transfer method. After blocking in 10% non-fat dried milk in TBST for 2 h, the blots were incubated with anti-Sox9 (Santa Cruz, diluted 1∶700) or anti-β-actin antibody (Santa Cruz, diluted 1∶1000) at 4°C overnight. β-actin acted as an internal control. After washing by TBST, the blots were incubated with a horseradish peroxidase-conjugated secondary antibody (Santa Cruz, diluted 1∶2000) at room temperature for 1 h. The blots were visualized by Femto (Pierce) following the manufacturer's instructions.

### Generation of Luciferase Reporter Constructs

Target sequences for a consensus miR-145-binding site (PT), two copies of the endogenous MREs sequence in the Sox9 mRNA 3′-UTR (MRE) and corresponding two copies of the MREs with a scrambled MREs sequence (MUT) were synthesized (Invitrogen) and cloned into the *Spe*I/ *Hind*III site of the pMIR-REPORT Firefly Luciferase reporter vector (Ambion) using standard DNA techniques. The correctness of three plasmid DNA constructs, shown as pMIR-PT, pMIR-MRE and pMIR-MUT, were further identified by sequencing. All target sequences of inserts are available in Table 1.

### Dual-luciferase Reporter Gene Assay

For luciferase activity analysis, HEK293 cells (2×10^5^ cells per well) were co-transfected with 125 ng of luciferase reporter constructs, 25 ng of phRL-TK (Promega) Renilla luciferase plasmid and 10 pmol of miRNAs with 1 µl Lipofectamine 2000 according to the manufacturer's instructions (Invitrogen). The miRNAs transfected into cells were purchased from Ambion, including pre-miR-145 precursor, anti-miR-145 inhibitor and their respective negative controls. After incubation for 48 h, we carried out the luciferase assay using dual-luciferase reporter assay system (Promega) per the manufacturer's instructions. Measurements of luminescence were performed on the luminometer (Glomax 20/20, Promega). In the dose-dependent experiment to explore the efficiency of miR-145 inhibition, 2.5 pmol, 5 pmol, 10 pmol of pre-miR-145 were used, respectively. Three independent experiments were performed, each time in triplicate.

### Alcian blue stain

To demonstrate the deposition of cartilage matrix proteoglycans, representative cultures were collected at indicated time points (day 3, day 7 and day 14) of induction and sulfated cartilage glycosaminoglycans (GAGs) were measured by alcian blue staining. The pellets for alcian blue staining were routinely fixed by 4% paraformaldehyde, dehydrated and paraffin imbedded. 5 µm sections were stained by 0.5% alcian blue 8GX (Amresco) for 20 min.

### Cell proliferation assay

Directly counting cells numbers from micromass pellets was used to detect the proliferation of C3H10T1/2 cells. The micromass pellets which were treated with either pre-miR-145 or anti-miR-145 inhibitor were induced to chondrocytes for 24 h. Subsequently, all micromass pellets were trypsinized by 0.25% trypsin and directly counted using a hemacytometer. All experiment were done in three independent experiments and repeated counting in triplicate.

### Statistical Analysis

Data are expressed as the mean±SD. Statistical comparisons were made between two groups with the *t*-test and between multiple groups with one-way ANOVA. A value of *P*<0.05 was considered significant unless otherwise described.

## Supporting Information

Figure S1 **Preliminary experiment of the non-transfected controls.** Pre-miR-145 (a final concentration of 50 nM), anti-miR-145 (a final concentration of 50 nM), their negative controls and non-transfected controls were transfected into C3H10T1/2 cells in 6-well pellets, respectively. After transfection, cells were induced to chondrocyte by TGF-β3 for 24 h and then harvested for measurement of Sox9 protein expression using Western blot. β-actin acts as an internal control. Quantitation of the Sox9 protein level was performed using Quantity One software. The result is shown in the below panels. There is no significant difference of Sox9 protein expression on cells between tansfected group and non-transfected control. Three independent experiments were done and data was represented as mean±sd. *, *p*<0.05, when compared with control.
(TIF)
