# Supplementary figures and images for: MicroRNA-145 Regulates Chondrogenic Differentiation of Mesenchymal Stem Cells by Targeting Sox9
Source: PLoS One. 2011 Jul 20;6(7):e21679. doi: 10.1371/journal.pone.0021679 (PMC3140487; doi:10.1371/journal.pone.0021679)

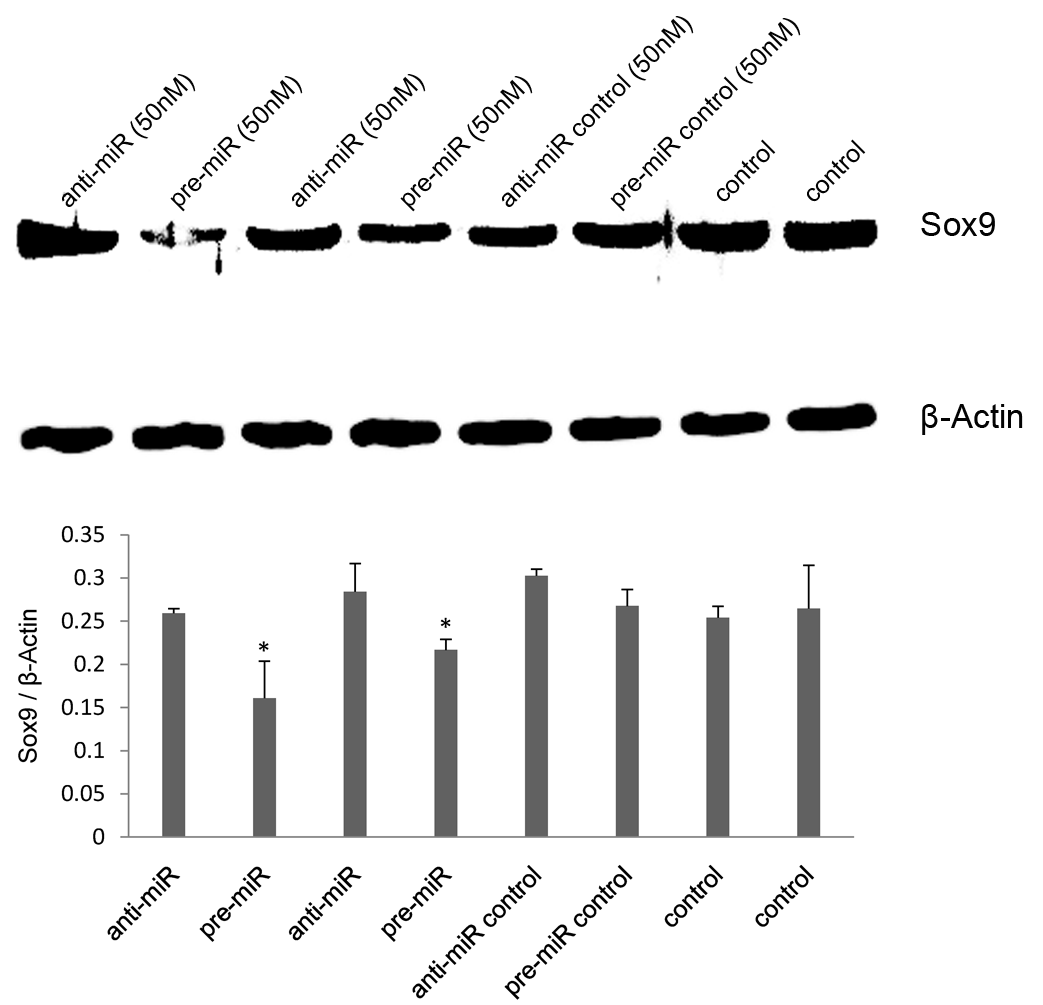

Supplement: Figure S1 — Preliminary experiment of the non-transfected controls. Pre-miR-145 (a final concentration of 50 nM), anti-miR-145 (a final concentration of 50 nM), their negative controls and non-transfected controls were transfected into C3H10T1/2 cells in 6-well pellets, respectively. After transfection, cells were induced to chondrocyte by TGF-β3 for 24 h and then harvested for measurement of Sox9 protein expression using Western blot. β-actin acts as an internal control. Quantitation of the Sox9 protein level was performed using Quantity One software. The result is shown in the below panels. There is no significant difference of Sox9 protein expression on cells between tansfected group and non-transfected control. Three independent experiments were done and data was represented as mean±sd. *, p<0.05, when compared with control. (TIF) [file pone.0021679.s001.tif]
